# Deep Learning Enables Automatic Correction of Experimental HDX-MS Data with Applications in Protein Modeling

**DOI:** 10.1021/jasms.3c00285

**Published:** 2024-01-23

**Authors:** Ramin E. Salmas, Antoni J. Borysik

**Affiliations:** Department of Chemistry, King’s College London, Britannia House, London SE1 1DB, U.K.

## Abstract

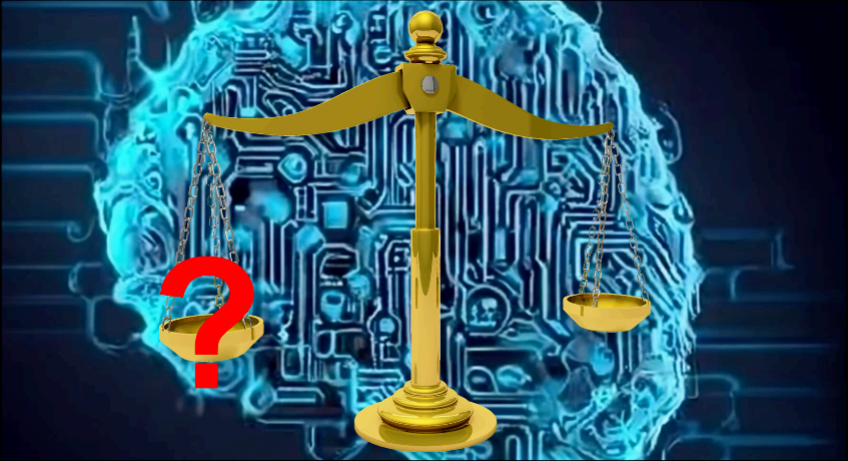

Observed mass shifts associated with deuterium incorporation in hydrogen–deuterium exchange mass spectrometry (HDX-MS) frequently deviate from the initial signals due to back and forward exchange. In typical HDX-MS experiments, the impact of these disparities on data interpretation is generally low because relative and not absolute mass changes are investigated. However, for more advanced data processing including optimization, experimental error correction is imperative for accurate results. Here the potential for automatic HDX-MS data correction using models generated by deep neural networks is demonstrated. A multilayer perceptron (MLP) is used to learn a mapping between uncorrected HDX-MS data and data with mass shifts corrected for back and forward exchange. The model is rigorously tested at various levels including peptide level mass changes, residue level protection factors following optimization, and ability to correctly identify native protein folds using HDX-MS guided protein modeling. AI is shown to demonstrate considerable potential for amending HDX-MS data and improving fidelity across all levels. With access to big data, online tools may eventually be able to predict corrected mass shifts in HDX-MS profiles. This should improve throughput in workflows that require the reporting of real mass changes as well as allow retrospective correction of historic profiles to facilitate new discoveries with these data.

## Introduction

Hydrogen–deuterium exchange mass spectrometry (HDX-MS) is an established biophysical technique to characterize protein structure and motion.^1^ The method exploits the natural exchange of covalently bound hydrogen atoms in proteins for deuterium in D_2_O solvent that result in time-dependent mass changes that can be followed by MS.^2,3^ Although all protons undergo exchange, the backbone amide protons occupy a particular exchange regime that allows their kinetics to be isolated from the remaining signals. Accordingly, HDX-MS offers a structural probe for every residue along the protein backbone excluding proline which cannot be detected due to the lack of an exchangeable NH. Protein exchange kinetics are characterized across a range of isotope exposure times and localized to different protein regions by acid proteolysis. A typical HDX-MS experiment yields numerous peptides of different lengths that can span the entire protein sequence with each peptide reporting mass shifts that accompanied exchange in solution.^4^ HDX-MS does not allow direct quantification of HDX kinetics for individual amino acids. However, the peptide mass increments provide qualitative feedback on dynamical variations between a perturbed and reference system such as those arising from a point mutation or additional of a ligand.^5^ Changes in protein dynamics can be mapped onto an available structure to illuminate aspects of protein function that can be invisible to more conventional structural biology.^6,7^ The complementary nature of HDX-MS along with its throughput, sensitivity, and capacity to work with virtually any protein system has allowed the technique to enjoy a decade of rising appeal.^8^

A peculiar feature of HDX-MS is that the isotope levels recorded experimentally invariably deviate from levels of deuterium incorporation obtained in solution. These exchange artifacts are nontrivial and not only compress the dynamic range of experimental data but alter its character.^9,10^ Isotope can be lost during protein digestion and chromatography such that peptides from flexible regions have the appearance of being more protected through a process of back exchange. Conversely, peptides from protected sites can undergo forward exchange by acquiring residual deuterium in quench solutions during proteolysis, making them appear less protected. Data can be corrected through the acquisition of appropriate controls, but thorough control analysis is frequently seen as unnecessary given the differential nature of conventional workflows where relative, not absolute, mass shifts are interrogated. Acquisition of data to control for back and forward exchange is also unappealing, as it requires additional sample preparation and extends all workflows as these data cannot be obtained outside of the primary experimental window. The main limitation of uncorrected HDX-MS data is its unsuitability for more advanced downstream processing such as optimization, which can be employed to model the underlying exchange kinetics. Uncorrected HDX-MS data are also incompatible with protein modeling approaches where data must conform to some form of predictive algorithm which cannot account for errors. Given that a significant number of historic data have been acquired without controls, considerable databanks exist for which more advanced processing is now impossible. The capacity to correct these data retrospectively is an interesting idea that could provide researchers with the potential to make new discoveries with existing data. The capacity to automatically correct HDX-MS data without the need to acquire experimental control data is also an appealing proposition that could entice a greater number of researchers to explore more advanced modeling of their data.

Here the possibility of using artificial intelligence (AI) to predict back and forward exchange control data from uncorrected HDX-MS profiles is demonstrated. Limited training data encompassing approximately 300 peptides from experimental HDX-MS acquisitions taken at 7 different isotope labeling times were used along with the respective experimentally determined back and forward exchange controls for each peptide. A model is trained using a multilayered perceptron (MLP) to map the different time point features to the back and forward exchange targets. MLP is a type of feedforward neural network (FFNN) among the deep neural networks (DNN) that has found particular applications in problems with unknown or challenging structure. The model is tested using a separate data set and various methods employed to gauge the overall accuracy of the AI generated data including various downstream processing tasks. Using this method, AI is shown to be able to correct back and forward exchange artifacts to within 5% of experimentally controlled data. The AI corrected HDX-MS data are also submitted for optimization and is shown to improve the accuracy of calculated HDX exchange kinetics by >35%. Finally, the ability of AI amended HDX-MS data to correctly identify native structures using an established modeling approach is investigated. The capacity of AI to improve HDX-MS guided modeling is remarkable, almost fully restoring the accuracy to levels expected for experimentally corrected data in classifying native protein folds. With eventual access to more substantial training data suitable for this application the potential for AI enabled automatic correction of HDX-MS data is clearly shown. Platforms could eventually become available for computational data correction enhancing throughput of experimental workflows and imparting new life into historic data.

## Materials and Methods

### Hydrogen–Deuterium Exchange Mass Spectrometry

The AI model was developed using previously obtained experimental HDX-MS data for enolase, serum amyloid P component, barnase, and α-lactalbumin.^11^ Data were acquired on a Synapt G2Si HDMS equipped with an Acquity UPLC M-Class system and automatic HDX-MS (Waters Corporation, Manchester, U.K.). For each protein, isotope uptake was recorded for 7 different exposure times between 15 s and 8 h with data analysis conducted using the ProteinLynx Global Server v3.0.2 and DynamX v3.0.0 (Waters Corporation, Manchester, U.K.). Separate data acquisitions were obtained for back and forward exchange controls of each protein. Briefly, all back exchange controls were obtained using fully exchanged protein samples with data acquired using a labeling time point of 15 s. Forward exchange controls were obtained for each protein using a reference, i.e., unlabeled acquisition, but with the quench buffer containing 50% D_2_O to match the ^1^H:^2^H ratio in the experimental quench solutions. After calculation of the relative fractions uptake (RFU) of all experimental time points and the control data, the experimental data sets of each protein were corrected for exchange artifacts.

### Characterization of Errors in HDX-MS Data

Relative fractional uptake (RFU) measurements for 100 back exchange (RFU_back_) and 100 forward exchange (RFU_fwd_) acquisitions were first taken from a pool of previously obtained experimental data. RFU_back_ represents residual deuterium remaining on peptides from fully exchanged protein samples following a 15 s HDX-MS labeling acquisition. These samples are not exposed to H_2_O until quenching and therefore contain information on the degree of isotope loss throughout the fluidics until ionization. RFU_fwd_ samples are used to determine the degree of isotope incorporation into peptides due to residual deuterium in the system. Since the final D_2_O concentration in quench solutions is 50% in our workflows, peptides from all protein regions, including those that are normally protected by the protein fold, undergo some artificial deuterium incorporation during the quench and online digestion. These measurements were acquired by obtaining reference HDX-MS acquisitions on ^1^H samples but with D_2_O added to the quench buffer. Properly collected RFU_back_ and RFU_fwd_ data for every peptide can be used to correct experimental data to the true RFU, i.e., the isotope level prior to quenching, digestion and chromatography.^2^ Histograms were then prepared from the pool of experimental RFU_back_ and RFU_fwd_ data and parameters from the fit of normal distributions used to prepare libraries of 10 000 back and forward exchange values. The effect of any error in back and forward exchange data on post processing was then characterized using a numerical approch that utilized synthetic data so that the introduction of error into RFU_back_ and RFU_fwd_ could be accurately tuned and the consequence of these errors on downstream processing clearly understood.

Experimental peptide maps were first taken for three different proteins. For these proteins the protection factors of each amino acid (ln *P*) were then simulated and used to build synthetic HDX-MS data (RFU) from the relevant polyexponential function as described previously.^12^ Back and forward exchange RFU was then assigned randomly to each of the peptides from our pool of data. The random assignment of these values to each peptide is justified by the lack of correlation between RFU_back_ and RFU_fwd_ in experimental data (Figure S2). The degree to which RFU_back_ and RFU_fwd_ altered the original synthetic RFU was then modeled according to eq 1 which is a rearrangement of the expression used to correct experimental data from back and forward exchange artifacts (eq 2).^2^ This process yielded RFU_err_ for each peptide in all three protein data sets which is the erroneous RFU of each peptide due to back and forward exchange, i.e., the masses following these artificial processes (eq 1).
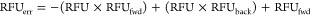
1At this stage, 4 different RFU values had been assigned to each peptide. These values included the original or true RFU, back and forward exchange RFU and RFU_err_ which is the change in the true RFU following back and forward exchange. The true RFU can be reconstituted from RFU_err_ using RFU_back_ and RFU_fwd_ according to eq 2. The next step was to characterize the degree to which variations in RFU_back_ and RFU_fwd_ would manifest in downstream processing following reconstitution of the data for each peptide. The RFU_back_ and RFU_fwd_ values were then varied according to 4 different thresholds at 2%, 5%, 10% and 20% RFU. These thresholds represented increasing degrees of experimental error in the acquisition of RFU_back_ and RFU_fwd_. Each variation was randomly assigned as either a positive or negative percent changeto prevent net drift in the RFU. Following the introduction of the variations in RFU_back_ and RFU_fwd_ the RFU_err_ of each protein was taken and the original RFU reconstituted for each peptide according to eq 2 using the modified RFU_back_ and RFU_fwd_ across all 4 thresholds.
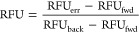
2

39 different HDX-MS profiles were prepared in total across the 3 different proteins including 12 data sets with increasing error thresholds in either the back or forward exchange RFU and these values in combination as well as an error free data set. These data were then submitted to HDXmodeller for optimization to model the underlying protection factors (ln *P*) of each amino acid.^12^ The effect of errors in RFU_back_ and RFU_fwd_ was then reported as the change in the coefficient of determination (Δ*R*^2^) with respect to the *R*^2^ between the error free data and the original reference ln *P* (Figure S1).

### Deep Learning Method

Training and testing of the algorithm was achieved using experimental HDX-MS data for 4 different proteins, including barnase, enolase, serum amyloid P component (SAP) and alpha lactalbumin. Data for barnase, enolase, and SAP, constituting ∼300 peptides, were used to train the model with data for α-lactalbumin, encompassing ∼80 peptides, used for validation. Each sample (peptide) consisted of 7 features including RFU values relating to different isotope exposure times and 2 targets comprising the respective back and forward exchange data. The system was trained to learn a map from the uncorrected RFU values of each peptide to the back and forward exchange RFU. For the purpose of generalizing the model and to enable it to handle input data with different time points, variance of the RFU was calculated to reduce the number of the features to a single value, eq 3 where *X* denotes the sample, *x* is the features (*x*_1_, *x*_2_, ..., *x*_*n*_), *n* is the total number of features, and μ is the feature average. Accordingly, the model will be able to correct RFU data of different samples irrespective of the number of time points or time point values.
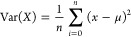
3

Feedforward neural network (FFNN) or multilayer perceptron (MLP), which is among the deep neural network (DNN) algorithms, was used to learn mapping between the input–output data.^13^ DNNs apply a polynomial transformation to the input features (*x*) replacing it with ϕ(*x*) (eq 4).
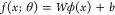
4In this linear form, the terms *W* and *b* relate to the weight and bias, respectively, and the parameter θ = (*W*, *b*) is updated during model development using gradient descent. The parameter ϕ(*x*) is a vector feature that is generated from the inputs and which takes the form shown below where *D* represents the degree of the model (eq 5).
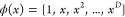
5To improve data learning capabilities, additional complexity is then introduced by adding the parameter θ_2_ as shown where θ = (θ_1_ + θ_2_) and θ_1_ = (*W*, *b*) (eq 6).
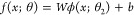
6The process of adding complexity continues until a function with high-dimensional characteristics (*f*_*L*_) is obtained (eq 7).
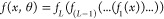
7It is instructive to note that perceptrons typically utilize deterministic logistic regression (eq 8)., where *w*^T^ is defined as *w*^T^*x* = *w*_1_*x* + *w*_2_*x* + *w*_D_*x* and *H* is a linear threshold function which imposes a linear boundary on the perceptron.^14^
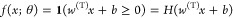
8Linear decision boundaries are frequently regarded as a limitation of perceptrons along with the nondifferentiable nature of H which precludes the use of gradient-based optimization for error minimization.^15^ The MLP approach was developed to address some of these limitations by structuring the training data into a vector represented by *x* ∈ *R*^*D*^ where *D* is the vector dimensions. The HDX-MS model used for this study was developed using 9 hidden layers and a layer rectified linear unit (ReLU) activation function which is classified as a nonsaturating activation function (eq 9).^16^ The number of layers used in the development of the model is a tunable parameter and was selected by monitoring performance while varying this parameter. Although a high number of layers are required to construct sophisticated models in deep learning, too many layers can result in overfitting. The density of the layers should therefore be seen as a hyperparameter that needs to be tuned.
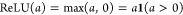
9

The ReLU function transforms negative values to zero but does not affect positive integers. A linear activation function was used in the output layer because of the requirement for the model output to take the form of continuous values. A mean squared error (MSE) cost function was used to calculate the difference between the predicted (*y̅*) and true values (*y*) as shown, where *D*_*i*_ denotes the node (*i*) (eq 10).
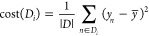
10

A well-known backpropagation or chain rule approach was used to generate the gradient of the cost function for the model’s output which was then employed for the gradient-descent optimization method.^17^ The difference in loss values between the true and prediction testing data set was determined to assess the predictive model’s uncertainty at each stage of the training process and determine if the model was over- or underfitted. It should be noted that the model learns only from the training data, such that the validation data can be seen as distinct for the purposes of further validation.

### Structural Modeling Using HDX-MS

A method based on the approach described by Karpus and co-workers was used to simulate protein protection factors (ln *P*) directly from single conformations.^18^ Data for α-lactalbumin was used as this has been shown previously to result in accurate structural predictions.^11^ Protection factors were used to generate HDX-MS data according to a polyexponential function as shown where n and t represent the number of amino acids in a peptide and experimental time point, respectively (eq 11).
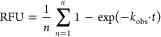
11

RFU was projected at various time points with proline and n-terminal residues discounted from the calculations. HDX-MS data were simulated for 1000 decoys structures prepared using 3D robot of which 83 structures yielded a backbone RMSD with the crystal structure of <2.5 Å and were labeled native.^19^ The correspondence (RMSE) between the simulated HDX-MS data was then determined for 3 samples including the corrected experimental data, uncorrected data, and AI-corrected data. A binary classification test was then performed to characterize the diagnostic ability of the HDX-MS simulations for native folds for each of the 3 samples. The test took the form of a receiver operating classification (ROC) plot from which the area under the curve (AUC) was used to quantify the test accuracy, which is taken as the ability of the test to correctly classify a native and non-native structure taken at random from the decoy set.

### Calculating Back and Forward Exchange from Random Coil Theory

The correct D–H or H–D correction terms were used to predict *k*_int_ for each amino acid in all peptides.^20^ For back exchange data, time was taken at the individual peptide retention times modified by 3 min for peptide trapping. Potential effects of acetonitrile on *k*_int_ were ignored since >80% of peptides eluted before 6 min where the concentration of acetonitrile was only 5% with the longest eluting peptide having a retention time of 6.4 min.^21^ Forward exchange calculations needed to factor in the rapid dilution of 50% D_2_O present in the quench. For this we took the average D_2_O concentration for 3 min trapping of 0.12% D_2_O. All rates were calculated at a pH 2.5 and at a temperature of 4 °C. Additional calculations were performed assuming a 30 s and 30 min transit through the pepsin column during which time rates were calculated at ambient temperature. For these calculations, the deuterium fraction of each backbone NH increased exponentially but would switch temperatures at the designated time. Rates were used to calculate the “D-incorporation” of each amino acid, from which the peptide RFU was established. RFU values were predicted assuming the spontaneous D–H conversion of the first and second amino acid in all peptides. The RFU was then used to correct the experimental HDX-MS profile and the outputs compared to experimental and AI corrected data.

## Results

Failure to adequately correct for back and forward exchange in HDX-MS can translate into errors with interpretation and further processing of the experimental data. The initial objective was to understand and quantify the impact of exchange artifacts on data optimization as an example of an advanced downstream processing task. Experimental RFU values deviate from the true values due to back and forward exchange, but the true RFU can be reconstituted by the acquisition of back and forward exchange data for each peptide (eq 2). While we expect uncorrected HDX-MS data to perform poorly with downstream processing such as optimization, we had no understanding of the tolerance of processing to differing degrees of error in back and forward exchange data. To understand this, a numerical approach was employed wherein the change in optimization accuracy was quantified in various synthetic data sets as errors were gradually introduced into the back and forward exchange RFU. Synthetic data were used in this instance as they allowed better isolation of the error effects and more certainty in quantifying the impact of back and forward exchange correction on the optimization process. HDX-MS data were built using 3 different peptide maps with isotope uptake projected in a polyexponential form from predetermined protection factors of each amino acid. Back and forward exchange data for each peptide were then selected at random from a pool of data produced in-house. Errors were then introduced into the control data according to different error thresholds between 2% and 20%. The synthetic HDX-MS data were then modified using the preselected back and forward exchange values and then reconstituted back to the original data but using the controls into which errors had been introduced. For each data set errors were generated for both the back and forward exchange RFU as well as each of the values in isolation and an error free sample. Overall, 39 data sets were prepared and submitted for optimization by HDXmodeller to model the underlying PFs of each amino acid and investigate the effect of errors in control data on the accuracy of the optimization process (Material and Methods, Figure S1).

**Figure 1:**
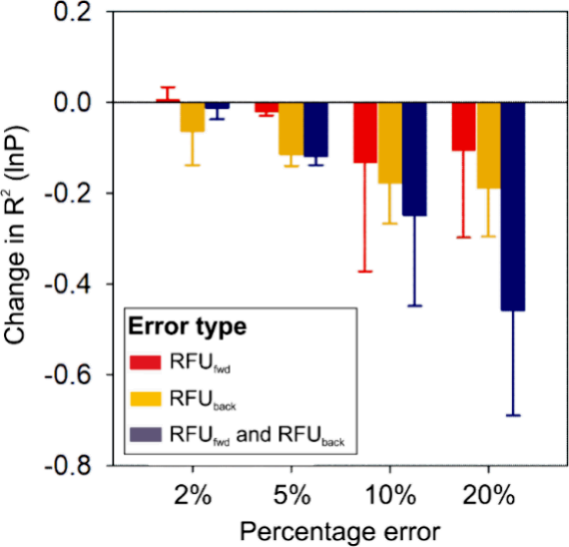
Effect of control errors on the accuracy of HDX-MS data optimization. Plot shows the change in *R*^2^ for optimized protection factors (ln *P*) in relation to the percentage error in control data. A total of 3 different data sets are represented in each bar along with the standard deviation. At each error threshold 3 different error types are shown representing errors in forward exchange data (red), back exchange errors (yellow), and errors in both back and forward exchange (blue).

As expected, a decrease in the accuracy (*R*^2^) of the modeled PFs was observed as the error in the back and forward exchange data increased from 2% to 20%. A predictable trend was also observed with data containing errors in both back and forward exchange values exhibiting the worst optimization outcomes and the best performing outputs belonging to those data for which errors were confined to the forward exchange values. In general, the impact of errors on the performance of optimization was considerable. Errors as little as 10% in both the back and forward control values translated to a reduction in the accuracy of modeled PFs of ∼0.25*R*^2^, increasing to >0.40*R*^2^ for errors of 20%. For data sets in which errors were isolated to forward exchange values only, the *R*^2^ reduced by >0.10 for errors as little as 10% (Figure 1). Given the variable nature of HDX-MS optimization across different data sets it is impossible to predict with high certainty exactly how erroneous or omitted control measurements will translate into errors with this processing task. However, it is clear that optimization is highly sensitive to even minor errors in HDX-MS control data such that the complete omission of controls will render any data set totally incompatible for this type of processing.

A model was then trained by using DNN to map uncorrected experimental HDX-MS data to the respective back and forward exchange control values of each peptide. The purpose of the model was to take experimental data for which no control measurements had been taken and predict the correct mass changes for each isotope expose time. The model was developed using limited training data constituting experimental HDX-MS profiles for 4 proteins including enolase, serum amyloid P component, barnase, and α-lactalbumin. Data for enolase, serum amyloid P component, and barnase comprising of ∼300 peptides were used to train the model with the remaining data set encompassing ∼80 peptides used separately for validation. All experimental data in the form of the relative fractional uptake (RFU) of each peptide can be downloaded from the example site of https://hdxsite.nms.kcl.ac.uk/Modeller in the “deep back” folder. All experimental data were acquired on a Synapt G2Si with automated HDX-MS along with appropriate control experiments for forward and back exchange correction acquired during the experimental window (Materials and Methods).

**Figure 2:**
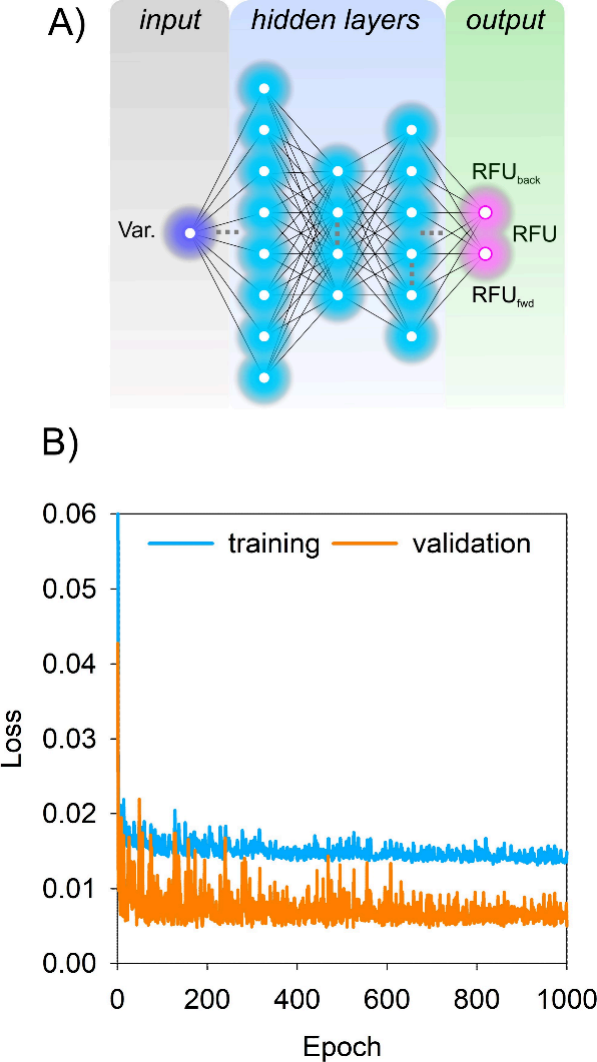
Overview of the NN and loss vs epoch graph for model generation. (A) Symbolic design of the NN used to map input–output data. The input was the variance (Var) of uncorrected RFU for all labeling time points taken as one-dimensional data. The NN included 9 hidden layers required to increase the dimensionality of the data. The output layer to which the data were mapped consisted of a linear function which generated continuous or real numbers for the corrected back (RFU_back_) and forward exchange (RFU_fwd_) data. (B) Loss outputs over 1000 epochs used to generate the model. The main metric for performance evaluation was the validation phase, with lower validation losses consistent with increasing confidence. The variance value used as input is the primary reason behind the model performing better in validation than in training.

A wide range of AI methods are available for this particular task including linear models, support vector machines (SVM), decision trees, ensemble methods, neural networks models (NN), and other nonlinear models that have been developed for mapping input-output data. Since each sample/peptide in the training set consists of targets or responses, which are continuous numbers, a supervised regression model was required for data mapping. The interpretability of the input-output mapping in some methods, including gradient boosting and DNN, is low due to the high complexity existing in the process. These types of “black-box” models contrast with other methods such as decision trees, where interpretability is high, but are frequently preferred due to their increased ability to generate accurate predictive functions. In this application interpretability was not a priority, and DNN were therefore employed since the principal task was to predict the corrected form of the data with no restrictions on the complexity of the mapping algorithm. The structure of the deep learning model involved 9 dense layers with different unit numbers on each layer. Uncorrected mass changes for each isotope exposure time expressed as the normalized or relative fractional uptake (RFU) were used as inputs with the RFU of the back and forward exchange controls representing the targets of the respective peptides. The NN was trained for a total of 1000 epochs with mismatch between the observed and predicted RFU reported through a loss function calculated using the mean square error (MSE). Error in the AI model was followed throughout the process until the model was deemed to have converged successfully with the validation process reporting a lower MSE suggesting the absence of any fundamental problems such as overfitting (Figure 2).

**Figure 3:**
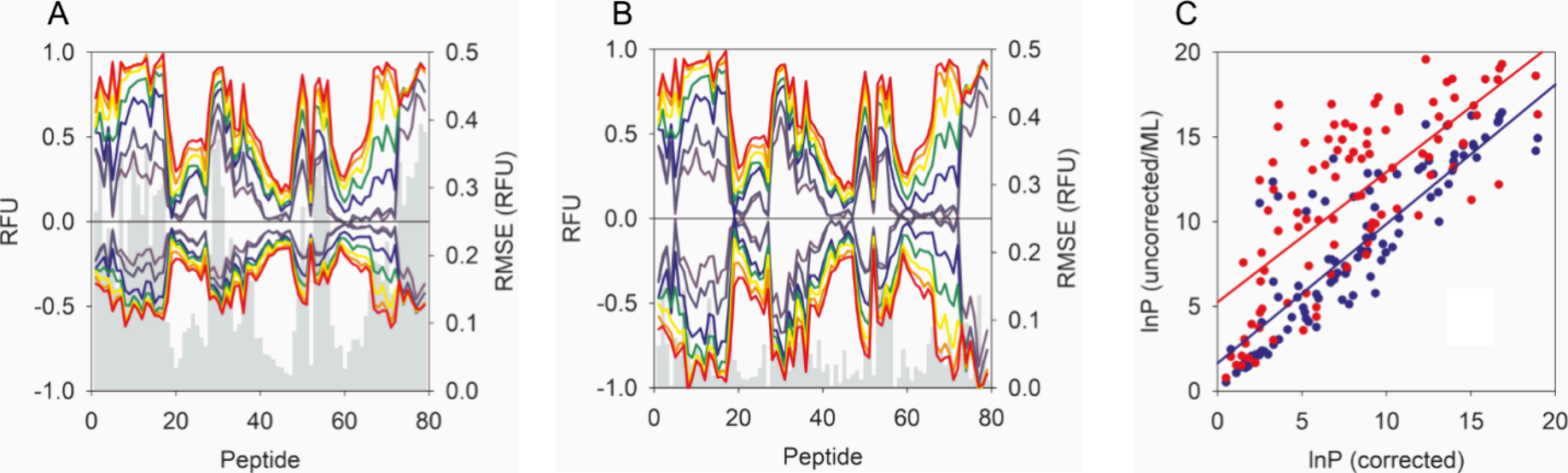
Ability of the AI model to correct for peptide and residue level HDX-MS data. (A) Comparison of experimentally corrected HDX-MS data for α-lactalbumin (positive frame) with experimental data lacking correction for back and forward exchange (negative frame). Data are represented as relative fractional uptake (RFU) across 7 different isotope exposure times from 15 s (violet) to 8 h (red). Gray bars represent the RMSE (RFU) between the corrected and uncorrected data for each peptide. (B) Data are represented as for (A) but with the RFU in the negative frame replaced with experimental data where the RFU has been corrected by AI. (C) Correspondence between protection factors, shown as the natural log of the protection factor (ln *P*), following optimization using HDXmodeller. Data show the correlation between ln *P* obtained from experimentally corrected RFU and the ln *P* from uncorrected data (red) and AI corrected data (blue) with respective *R*^2^ values of 0.41 and 0.77.

The ability of the model to accurately correct HDX-MS data was then quantified using the supplementary data set not used for training. This data set was highly corrupted due to back and forward exchange with errors ranging from 0.03 to 0.40 RMSE (RFU) for individual peptides and 0.197 RMSE (RFU) for the entire data set. It is instructive to restate that no method currently exists that can restore HDX-MS data to their corrected values other than the experimental acquisition of back and forward exchange data for each peptide. Single probe methods to follow the extent of erroneous exchange are ineffective as they wrongly assume that all peptides behave as random coils under quench conditions.^9^ Levels of back and forward exchange for individual peptides are also uncorrelated, making it impossible to predict the error in one direction from values determined experimentally for the other. Remarkably the AI model is capable of almost fully restoring the uncorrected data to the correct values, reducing the overall error from 0.197 RMSE to 0.05 RMSE (RFU). To provide a more rigorous test of the model, data constituting the uncorrected profile along with the data sets corrected both experimentally and by AI were all submitted for optimization to model the underlying PFs. As expected, PFs obtained for the uncorrected data correlated poorly with the experimentally corrected data with a coefficient of determination (*R*^2^) between the outputs of only 0.41. In contrast, PFs modeled using the AI-corrected data achieved a correlation (*R*^2^) of 0.77 demonstrating a marked increase in the fidelity of these data. AI is therefore able to enhance data accuracy at different levels and demonstrates considerable potential for automatic HDX-MS data correction that can be expected to improve as more training data become available (Figure 3).

To further gauge the accuracy of the model, we then compared the AI corrected data to data corrected using predictions of back and forward exchange from HDX theory. Calculating peptide back exchange is complicated, as it assumes that tabulated D-H or H-D correction factors for aqueous solutions hold in MS buffers containing organic solvent. While reasonable estimates of time can be made from chromatographic peptide retention modified by trapping time it is difficult to establish transit times during digestion where HDX rates are governed by ambient temperatures unlike the remainder of the chromatographic process where temperature are held at 4 °C. Efforts to calculate back exchange have been made previously but require the use of internal probes for normalization which contradicts the probe-free premise of this work.^22^ Predictions of back and forward exchange must also incorrectly assume random coil conformations and overlook column interactions which can vary the rates unpredictably.^9^ In spite of these challenges, calculations of the degree of back and forward exchange were made for the system. These predictions were then used to modify the uncorrected data, and the results were compared to data corrected by experiment or by AI (Materials and Methods). Direct comparison of RFU calculations for back and forward exchange data to those predicted by AI were not possible owing to the complex nature of the MLP. In all peptides, back exchange calculations drastically underestimated the degree of isotope loss post quench resulting in a considerable reduction in the dynamic range of corrected data and an overall RMSE of 0.16 RFU against experimental corrections. Assuming spontaneous D–H conversion of amino acids in the second position, all peptides improved the correlation but were still insufficient at accurately mimicking the extensive back exchange in the system (RMSE 0.11). To address this, the temperatures of all calculations were modified assuming all peptides underwent 30 s transit at ambient temperatures during chromatographic digestion. This modification has little effect on the predicted data and so was extended to 3 min which is the maximum time any peptide can persist in the digestion column. This change brought the calculated HDX-MS profile more in line with experimentally corrected data with a total RMSE of 0.085 but was still inferior to corrections made by the AI model. Corrections based on calculated methods were also less able to capture the overall character of the HDX-MS profile compared to AI. Overall these calculations highlight the challenges of predicting back and forward exchange in the absence of an internal standard (Figure S3).

**Figure 4:**
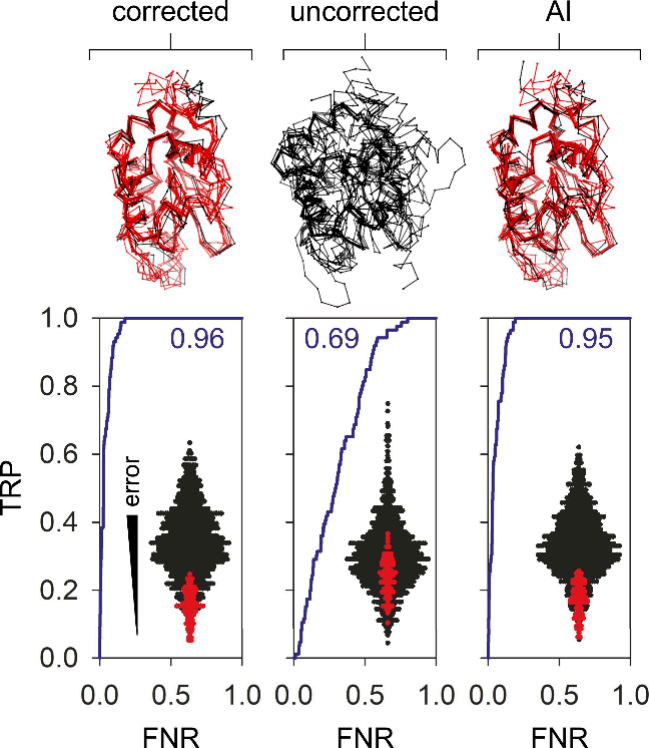
AI generated data allow conformational selection using HDX-MS. Receiver operating characteristic (ROC) plots summarize the ability of HDX-MS data to identify native protein folds of α-lactalbumin in a binary classification task. Plots show the relationship between the false positive rate (FPR) and true positive rate (TPR) across various thresholds for experimentally corrected data, uncorrected data, and AI corrected data, shown left to right. The area under the curve (AUC) is reported which is akin to the accuracy of each test with a value of 0.5 consistent with a random outcome. Data were generated using 1000 decoy structures represented by the inserted dot plots showing the density of different folds arranged according to the correspondence of their respective HDX-MS simulations with experimental data. Structures represent an alignment of the top 10 folds selected on the basis of HDX-MS data simulations. Red and black in the dot plots and structures represent native and non-native folds, respectively.

The ability of the AI corrected data to be used for protein modeling applications was then investigated. While methods to exploit HDX-MS for *ab initio* protein structure determination have yet to be established certain approaches have demonstrated effectivness with specific systems. We previously quantified the ability of a popular phenomenological method to model proteins folds using an unbiased “sample and select” modeling approach. This method was shown to be capable of identifying the native fold of alpha lactalbumin from 1000 decoy states with an accuracy of >96% based on an experimental HDX-MS profile of the protein.^11^ In this approach, HDX-MS data were simulated for all decoys based on the phenomenological method and the structures ranked based on the similarity of their associated HDX-MS profiles with an experimentally obtained data set of the protein. Accuracy was assessed from an outputted receiver operating characteristic (ROC) plot, reporting the probability of correctly classifying a native and a non-native structure taken at random from each class. Replacing the experimentally corrected HDX-MS data of α-lactalbumin for a data set without correction reduces the accuracy of modeling this system to just 69%. This loss of accuracy highlights the ineffectiveness of using uncorrected HDX-MS data for protein structure determination and cautions against using these data for this particular application. However, the AI model is capable of almost fully restoring the ability of HDX-MS to modeling this structure yielding an accuracy of 95% (Figure 4). This demonstrates a highly compelling aspect of AI through its potential to rescue previously acquired data sets, for which no control measurements were acquired, and provide these data with access to new applications in structure determination.

## Conclusion

Continual advancements in AI will open up a multitude of new applications, and the transformational potential of the technology cannot be underestimated. Research should anticipate a surge in the use of AI bringing an associated increase in throughput, accuracy, and simplicity to most areas. Here we report a unique application of deep learning to amend errors in experimental HDX-MS profiles arising from back and forward exchange artifacts that compress and vary the true signal. The AI model has been developed using a sparce training set consisting of only ∼300 peptides, and more accurate models should be anticipated with eventual access to big data. The utility of this application is demonstrated through a remarkable ability to improve the fidelity of uncorrected data and enhance the accuracy of outputs obtained from various advanced processing tasks. As more processing tools for HDX-MS become available, allowing deeper insights into protein function, the requirement for complete data correction is likely to increase. Currently, the only viable method to restore HDX-MS mass shifts to their correct values is through the acquisition of experimental control data to account for back and forward exchange. While experimental methods are likely to remain the most reliable approach, AI may provide an appealing alternative for workflows that prioritise throughput. Perhaps the most attractive application of this method lies with historic data sets where control measurements have been omitted. These databanks are likely to be considerable, and the requirement for controls to be collected within the experimental window may render these data incompatible with many further processing tools. In these scenarios, the ability to retrospectively apply mass correction using AI should offer immediate appeal, allowing new meaning and discovery to be gained from surplus data.

## Data Availability

The codes for the deep neural networks can be obtained from GitHub https://github.com/raminsalmas/DeepBack. Experimental data in the form of the RFU of each peptide can be downloaded as text files from the example site of https://hdxsite.nms.kcl.ac.uk/Modeller in the “deep back” folder.

## Supporting Information Available

Figure S1 showing summary diagram of the process used to introduce and characterize errors in HDX-MS data; Figure S2 showing plot of the relationship between experimental back and forward exchange data; Figure S3 showing different methods to correct back and forward exchange (PDF)
