# Supplementary material for: Deep Learning Enables Automatic Correction of Experimental HDX-MS Data with Applications in Protein Modeling
Source: J Am Soc Mass Spectrom. 2024 Jan 23;35(2):197–204. doi: 10.1021/jasms.3c00285 (PMC10853964; doi:10.1021/jasms.3c00285)
Supplement: Supplementary file 1 — js3c00285_si_001.pdf [file js3c00285_si_001.pdf]

Supporting Information

Deep learning enables automatic correction of experimental HDX-MS data with applications in protein modelling.

Ramin E. Salmas and Antoni J. Borysik*

antoni.borysik@kcl.ac.uk

Department of Chemistry, Britannia House, King's College London SE1 1DB UK

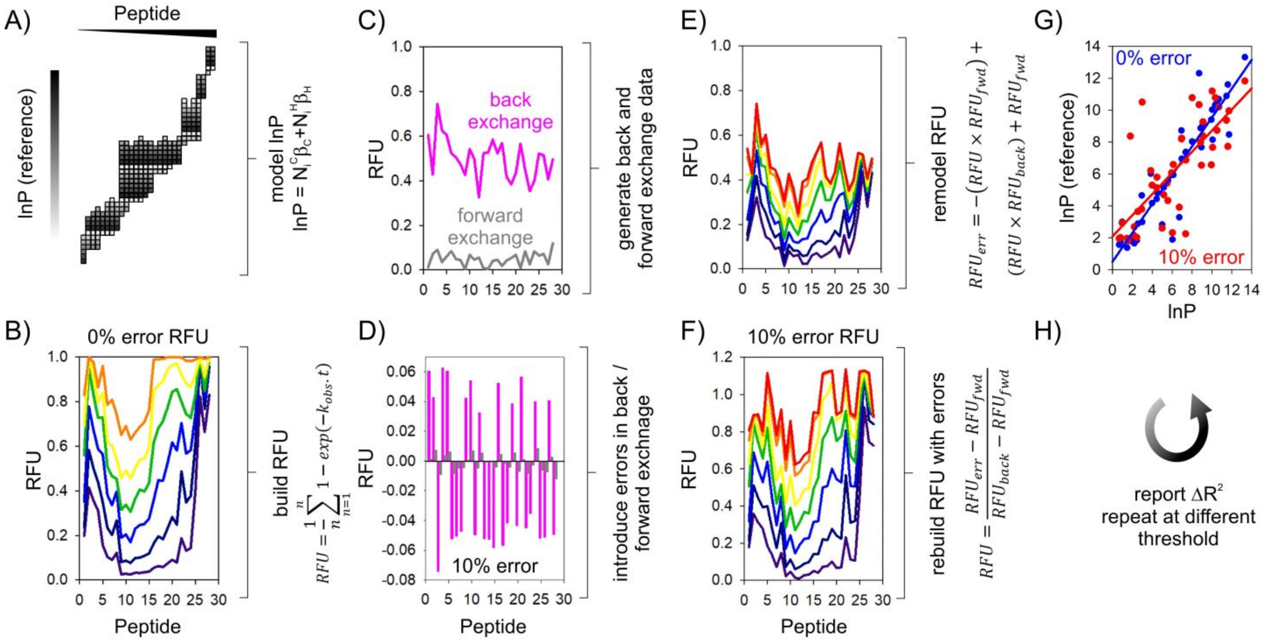

Figure S1: Overview of process used to introduce errors in HDX-MS data. **(A)** Experimental peptide maps were taken, and reference protection factors (lnP) simulated for each amino acid directly from the relevant crystal structure using the phenomenological estimation as shown. 3 different peptide maps were used in total and the accuracy of the simulated reference lnP was not relevant for this application. **(B)** HDX-MS data were built for each peptide map using the reference lnP in conjunction with a polyexponential function, as shown. N-terminal amino acids and proline residues were omitted from these calculations. **(C)** Back and forward exchange RFU were taken at random for each peptide from a library of values. **(D)** Each of the back and forward exchange values were then adjusted according to a different error threshold between 2% and 20% RFU with identical adjustment made for each of the 3 peptide maps. The sign of the associated error values was applied at random to each peptide to avoid net drift in the data. **(E)** Back (RFU_{back}) and forward (RFU_{fwd}) exchange artefacts were then introduced into the 0% error HDX-MS data **(B)** to remodel the RFU as shown. **(F)** The original RFU_{back} and RFU_{fwd} data **(C)** were then replaced for the control RFU into which errors had been introduced **(D)** and the RFU rebuilt to introduce these errors. **(G)** All HDX-MS data, including the 0% error projections and the data containing errors prepared at different thresholds, were then submitted for optimisation to model the underlying lnP using HDXmodeller. The change in the R^2 (ΔR^2) was then reported by calculating the change in correspondence between the model and the original reference lnP. **(H)** This procedure was repeated for data at each error threshold and the mean and standard deviation of the (ΔR^2) reported across the 3 peptide maps.

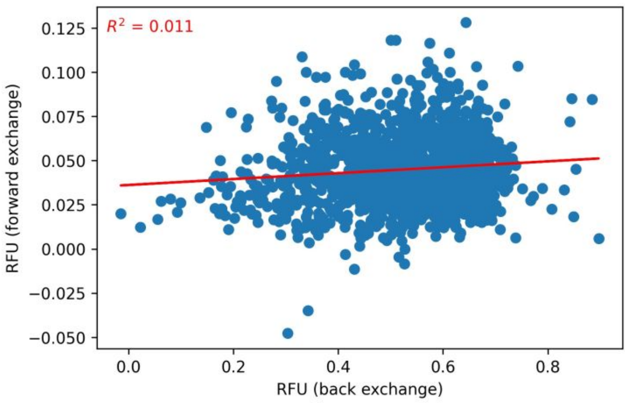

Figure S2: Relationship between back and forward exchange data. Plot shows the correlation between the back and forward exchange RFU for > 1600 experimental peptides. Back exchange data represent residual RFU for fully exchanged proteins samples with data acquired as a 15 second labelling experiment. Forward exchange data represent isotope gained for protonated protein samples using a reference acquisition with quench solutions containing 50% D_2O. Note the poor correlation between the data (R^2 = 0.011) highlighting the inability of predicting one of the terms from either of the experimental values.

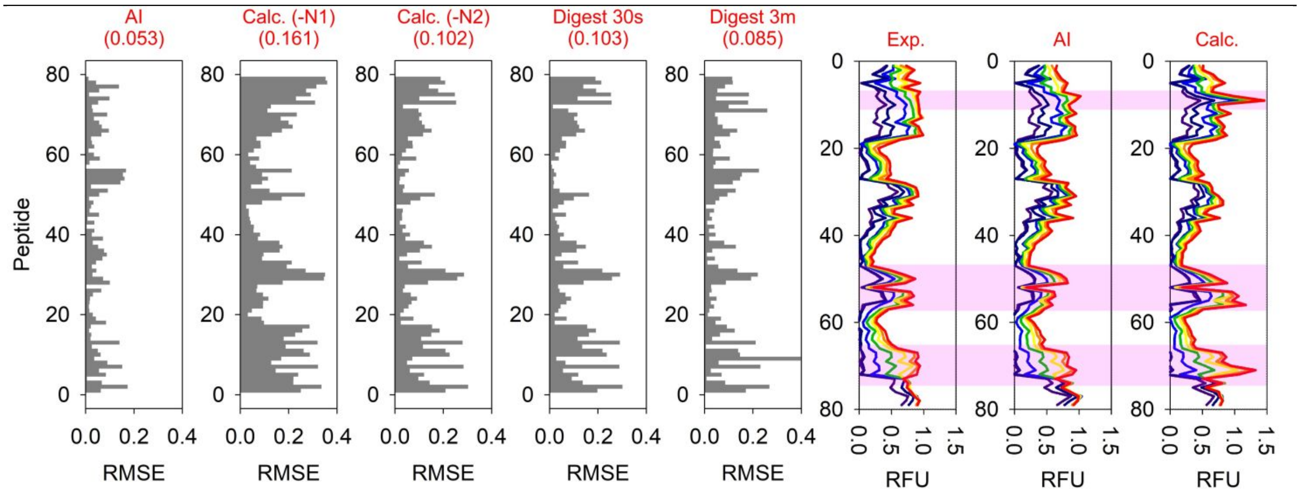

Figure S3: characterisation of different methods to correct back/forward/exchange. Bar charts show the RMSE (RFU) between peptides corrected experimentally and those corrected using AI and various calculated methods based on first principles including, predictions made assuming spontaneous exchange of the first one (-N1) and first two (-2N) amino acids and predictions made assuming 30 second (30s) and 3-minute (3m) transit during digestion using -2N data. The total RMSE (RFU) is given for each plot in parentheses. The plots on the right shown show the relative fractional uptake (RFU) of the same peptides over all 7 timepoints from 15 seconds (violet) to 8 hours (red). Plots show data corrected by experiment (Exp.), artificial intelligence (AI), and data corrected using calculations assuming 3-minute (3m) transit during digestion. Note how the calculated method fails to predict the character of the corrected HDX-MS data in several regions particularly those highlighted in pink.
